# Are high school girls’ lacrosse players at increased risk of concussion because they are not allowed to wear the same helmet boys’ lacrosse players are required to wear?

**DOI:** 10.1186/s40621-020-00242-5

**Published:** 2020-05-18

**Authors:** R. Dawn Comstock, Alan T. Arakkal, Lauren A. Pierpoint, Sarah K. Fields

**Affiliations:** 1 Department of Epidemiology, Colorado School of Public Health, University of Colorado Anschutz, 13001 E. 17th Place, Mail Stop B119, Fitzsimons Building, Room W3145, Aurora, CO 80045 USA; 2 Department of Epidemiology, College of Public Health, University of Iowa, Iowa City, IA USA; 3 Steadman Philippon Research Institute, Vail, CO USA; 4 Department of Communication, University of Colorado Denver, Denver, CO USA

**Keywords:** Concussion, Lacrosse, Gender, Attributable risk, Attributable risk percent, Surveillance, Prevention

## Abstract

**Background:**

Boys’ lacrosse (LAX), a full contact sport allowing body and stick checking, mandates hard shell helmets with full face masks. Girls’ LAX, which prohibits body checking and whose sphere rule is supposed to prevent stick checking to the head, allows optional flexible headgear with/without integrated eye protection. Whether the required boys’ LAX helmets should also be mandated in girls’ LAX has been debated.

**Methods:**

In this retrospective cohort study we used LAX concussion data from a national high school sports-related injury surveillance study to determine if girls’ LAX players were at increased risk of concussion from stick or ball contact due to differences in helmet regulations by calculating the attributable risk and attributable risk percent (AR%) for concussion resulting from ball or stick impacts.

**Results:**

From 2008-09 through 2018–19, boys’ LAX players sustained 614 concussions during 1,318,278 athletic exposures (AEs) (4.66 per 10,000 AEs) and girls’ LAX players sustained 384 concussions during 983,291 AEs (3.91 per 10,000 AEs). For boys, athlete-athlete contact was the most common mechanism of concussion accounting for 66.4% of all concussions, while stick or ball contact accounted for 23.5%. For girls, stick or ball contact accounted for 72.7% of all concussions, while athlete-athlete contact accounted for 19.8%. Concussion rates from stick or ball contact were significantly higher in girls vs. boys (RR = 2.60, 95% CI 2.12–3.18). The attributable risk associated with playing girls’ vs. boys’ LAX for concussion resulting from stick or ball contact was 1.75 concussions per 10,000 AEs (95% CI 1.37–2.12) and the AR% was 61.5% (95% CI 52.9–68.5). An estimated 44.7% of all girls’ LAX concussions could have been prevented if girls’ LAX players wore the helmet mandated in boys’ LAX.

**Conclusions:**

Girls’ LAX players who are allowed, but not required, to wear a flexible headgear are at increased risk of concussions from stick or ball impacts compared to boys’ LAX players, who are required to wear a hard shell helmet with full face mask. Additional research is needed to determine if there are any defendable arguments to continue justifying restricting girls’ LAX players access to this effective piece of protective equipment.

## Background

High School Lacrosse (LAX) has experienced rapid growth in the United States (US) over the past decade. Between 2008-09 through 2018–19, participation in boys’ LAX rose from 88,596 athletes in 1984 schools to 113,702 athletes in 3026 schools, while participation in girls’ LAX rose from 64,929 athletes in 1780 schools to 99,750 athletes in 2877 schools (NFHS High School Participation Survey Archive, 2019a). Like most school sports, LAX concussion rates have increased over time (Marar et al. 2012; Xiang et al. 2014; Pierpoint et al. 2019a, 2019b). A study comparing concussion rates across 20 high school sports found that among boys’ sports, LAX had the third highest concussion rate following football and ice hockey, while among girls’ sports, LAX had the highest concussion rate just above soccer (Marar et al. 2012). A more recent study found that although concussion rates were slightly higher in boys’ vs. girls’ LAX (4.8 per 10,000 vs 4.0, RR = 1.2, 95% CI 1.0–1.4), concussions represented a nominally greater percentage of all injuries in girls’ LAX (23.1 vs. 25.6%) (Warner et al. 2018). This similarity across genders is surprising given several rule differences that should protect girls’ LAX players from concussion.

Although the objective of LAX (throwing a ball into a goal using a stick with a webbed head pocket) and many of the physical actions (e.g., rapid acceleration, deceleration, changes of direction, throwing, catching, and cradling a hard, fast moving ball) are the same, there are several substantial rule differences (US Lacrosse n.d.-a). Boys’ LAX is a full contact/collision sport that allows both body checking and stick checking. Conversely, in girls’ LAX body checking is prohibited, and although stick checking is allowed, a “halo”/sphere rule defines an imaginary sphere of 7 in. surrounding the player’s head in all directions that is not to be breached. However, the sphere rule is not always enforced. One study reported illegal stick and body contacts to the head in girls’ LAX competitions rarely resulted in a penalty (Caswell et al. 2017). A US Lacrosse Coaches Health & Safety Rules article, “Understanding the Women’s Safety Sphere,” noted this lapse, concluding “The rules, as they are written, are not always as they are played, coached, or officiated” (Kelley 2018).

**Fig. 1:**
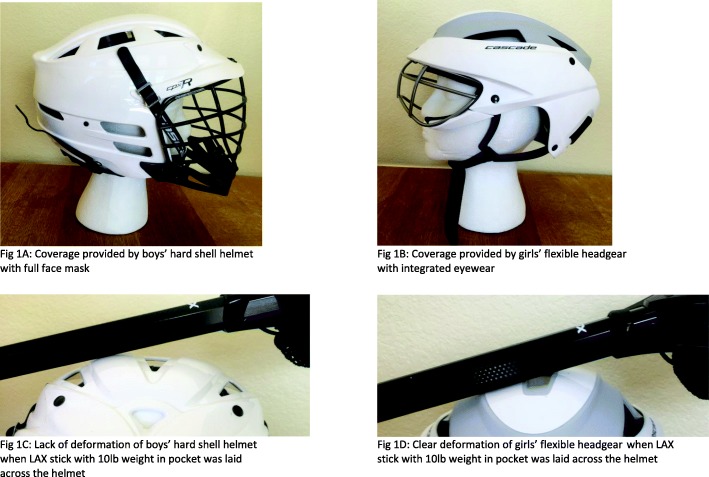
Examples of the Hard Shell Helmets Mandated in Boys’ LAX and Optional Flexible Headgear Allowed in Girls’ LAX. **a**: Coverage provided by boys’ hard shell helmet with full face mask. **b**: Coverage provided by girls’ flexible headgear with integrated eyewear. **c**: Lack of deformation of boys’ hard shell helmet when LAX stick with 10lb. weight in pocket was laid across the helmet. **d**: Clear deformation of girls’ flexible headgear when LAX stick with 10 lb. weight in pocket was laid across the headgear. Note: **c** and **d** document the same static, weighted lacrosse stick resting at an angle across a never worn male helmet and a never worn female headgear. There was no stick strike (e.g., no acceleration of the stick toward the helmet/headgear) when the photos were taken

Boys’ LAX rules require players wear hard shell helmets with full face masks (Fig. 1), mouth guards, shoulder/arm pads, and padded gloves. In contrast, because girls’ LAX rules are supposed to protect against head injury, girls’ LAX players are only required to wear mouth guards and protective eyewear and are explicitly prohibited from wearing the hard shell, full face masked helmet mandated in boys’ LAX. Although soft headgear not specifically designed for LAX (e.g. rugby scrumcaps, soccer headbands) were allowed, there was no standard for a women’s LAX headgear until ASTM released performance standard F3137–15, Standard Specification for Headgear Used in Women’s Lacrosse (excluding Goalkeepers) (ASTM F3137-15 2015). A women’s LAX headgear was not marketed until 2016. Subsequently, US Lacrosse released a point of emphasis update on the newly optional, but not required, women’s lacrosse headgear (Fig. 1), declaring any headgear used after January 1, 2017 must meet the new ASTM standard (US Lacrosse n.d.-a), which mandates a flexible shelled headgear and explicitly not a hard shelled helmet. In this document US Lacrosse stated, “The headgear standard was developed to decrease ball-to-head and stick-to-head impact forces.”

As expected, given the differences in LAX rules and protective equipment, mechanisms of concussion differ across genders. Several studies have reported the most common mechanism of concussion in boys’ LAX is athlete-athlete contact, while the most common mechanism among girls’ LAX is stick or ball contact (Lincoln et al. 2007; Xiang et al. 2014; Warner et al. 2018; Pierpoint et al. 2019a, 2019b). Ball contact has been found to be associated with the highest head impacts in girls’ LAX (Caswell et al. 2017). The hard shell helmets mandated in boys’ LAX have been demonstrated to be effective at reducing head injuries from potential ball strikes (Rodowicz et al. 2015). Yet, for nearly three decades whether the helmets required in men’s/boys’ LAX should also be required in women’s/girls’ LAX has been debated (Lapidus et al. 1992; Harmer 1993; Diamond and Gale 2001; Otago et al. 2007; Dick et al. 2007; Lincoln et al. 2007; Xiang et al. 2014; Warner et al. 2018; Pierpoint et al. 2019a, 2019b).

A recent clinical review of manuscripts reporting women’s LAX injuries published since 1950 found that women sustained higher percentages of head and facial injuries than men and women’s injuries occurred most frequently from stick or ball contact rather than athlete-athlete contact (Barber Foss et al. 2018). Thus, we hypothesized in this study that playing boys’ LAX, where hard shell helmets are mandated, was protective against concussion from stick or ball impacts, while playing girls’ LAX, where such helmets are prohibited, was a risk factor. Our objective was to use LAX concussion data from a large national high school sports-related injury surveillance study to determine if girls’ LAX players were at increased risk of concussion from stick or ball contact because they are not allowed to wear the same helmet mandated in boys’ LAX. Our specific aims were to calculate and interpret the attributable risk and attributable risk percent (AR%) for concussion resulting from ball or stick impacts associated with the different gendered helmet rules.

## Methods

### Data source and study definitions

In this retrospective cohort study, we analyzed National High School Sports-Related Injury Surveillance Study (High School RIO) data collected during the 2008–09 through 2018–19 academic years (High School RIO n.d.). The methodology of High School RIO has been described previously (Rechel et al. 2008; Kerr et al. 2018). In brief, High School RIO captures injury and athletic exposure (AE) data from a large sample of US high schools annually. Eligible participants are US high schools with National Athletic Trainers’ Association (NATA)-affiliated certified athletic trainers (ATs) who have valid e-mail addresses and are willing to participate in the study. Weekly throughout each sport’s season, ATs complete injury and exposure reports via a secure website. For each injury the AT completes a detailed injury report, providing information on the injured athlete (age, position played, etc.), the injury (body site, diagnosis, etc.), and the injury event (competition vs. practice, mechanism, etc.). Throughout each academic year ATs were able to view and update previously submitted reports as needed with new information (time loss, etc.). High School RIO began capturing data on 9 original sports of interest in the 2005–06 academic year, but boys’ and girls’ LAX were not included until High School RIO expanded to add a convenience sample of US high schools during the 2008–09 academic year. In this study, we analyzed concussion injuries sustained in girls’ and boys’ LAX. Although High School RIO captures whether or not girls’ LAX players who sustained head injury were wearing optional headgear, uninjured athletes’ use/non-use of this protective equipment is not captured.

In High School RIO, a LAX concussion injury was defined as an injury that 1) occurred as a result of participation in a school-sanctioned organized practice or competition and 2) required medical attention by a certified AT or physician. An AE was defined as one student-athlete participating in one school sanctioned competition or practice during which he or she was exposed to the possibility of athletic injury, regardless of the time associated with that participation. High School RIO reports AEs for competition and practice. High School RIO categories for LAX specific mechanisms of injury were collapsed as follows: stick or ball contact, athlete-athlete contact, and “other” which consisted of all other mechanisms including contact with playing surface, contact with goal, unknown, etc. Stick and ball contact were collapsed into one category for this evaluation of the gendered helmet regulations because helmets are known to be effective in reducing forces from both ball and stick strikes and as US Lacrosse has stated regarding the new female helmet, “The headgear standard was developed to decrease ball-to-head and stick-to-head impact forces.” (US Lacrosse n.d.-a).

### Statistical analysis

Data were analyzed using SAS, version 9.4 (SAS Institute Inc., Cary, North Carolina). We examined concussion counts and distributions by event type (practices and competitions) and mechanism of injury. Concussion rates per 10,000 AEs and rate ratios (RRs) for gender comparisons using boys’ LAX as the referent category were calculated. All RRs with 95% confidence intervals (CIs) not containing 1.0 were considered statistically significant. We used linear regression to analyze linear trends of concussion rates over time.

Concussion risk in girls’ and boys’ high school lacrosse are multifactorial and mechanisms of concussion differ across genders (Lincoln et al. 2007; Xiang et al. 2014; Warner et al. 2018; Pierpoint et al. 2019a, 2019b). Thus we calculated the attributable risk and AR% to assess only those concussion resulting from stick or ball impacts associated with the different gendered helmet rules. Attributable risk, the rate of the outcome among those exposed that can be attributed to the exposure, is calculated by subtracting the rate of the outcome among the unexposed from the rate among the exposed. Thus, we subtracted the rate of concussions sustained from stick or ball contact in boys’ LAX from the rate of concussions sustained from stick or ball contact in girls’ LAX to estimate the rate of concussions resulting from stick or ball impacts that could have been prevented if girls’ LAX players (the exposed) wore the hard shell helmets mandated in boys’ LAX (the unexposed). The AR%, the percent by which the incidence rate of the outcome among those exposed would be reduced if the exposure were eliminated, was calculated as [(incidence rate in the exposed – incidence rate in the unexposed)/incidence rate in the exposed)]*100. We estimated the total percent of girls’ LAX concussions that could be prevented if girls’ LAX players wore the same helmet mandated in boys’ LAX by multiplying the AR% for concussion resulting from stick or ball impacts by the overall percent of girls’ LAX concussions sustained as a result of stick or ball contact. The High School RIO surveillance study was approved by Nationwide Children’s Hospital Subjects Review Board (Columbus, OH) and Colorado Multiple Institutional Review Board (Aurora, CO).

## Results

### Concussion rates by type of activity and over time

**Fig. 2:**
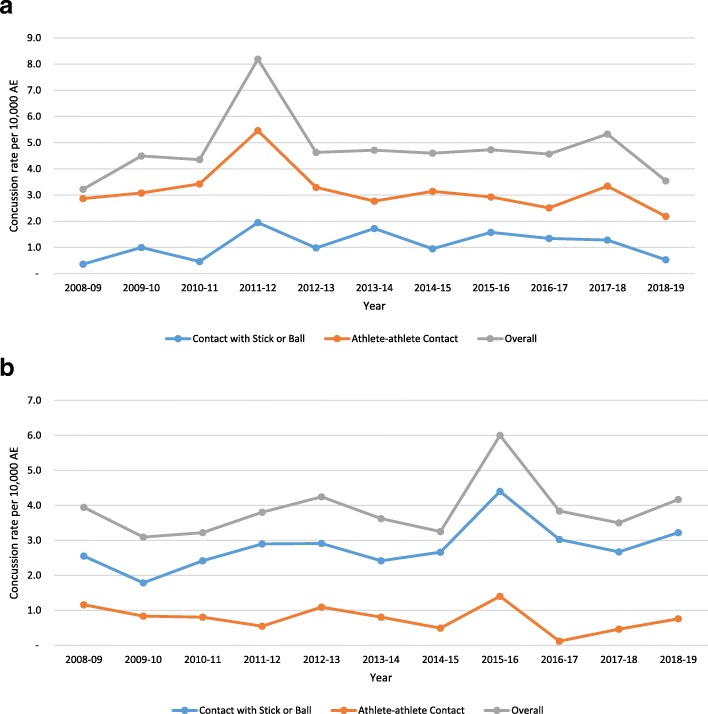
**a** Boys’ Lacrosse Concussion Rates Over Time by Injury Mechanism, The National High School Sports-Related Injury Surveillance Study, 2008–09 Through 2018–19. *p*-value level of significance, *p* < 0.05. *p*-values for trends over time: Contact with Stick or Ball, *p* = 0.51; Athlete-Athlete Contact, *p* = 0.26; Overall *p* = 0.92. **b** Girls’ Lacrosse Concussion Rates Over Time by Injury Mechanism, The National High School Sports-Related Injury Surveillance Study, 2008–09 Through 2018–19. *p*-value level of significance, *p* < 0.05. *p*-values for trends over time: Contact with Stick or Ball, *p* = 0.09; Athlete-Athlete Contact, *p* = 0.26; Overall *p* = 0.37

From 2008-09 through 2018–19, boys’ LAX players sustained 614 concussions during 1,318,278 AEs (4.66 per 10,000 AEs) and girls’ LAX players sustained 384 concussions during 983,291 AEs (3.91 per 10,000 AEs). The concussion rate was significantly higher in boys’ compared to girls’ LAX (RR = 1.19, 95% CI 1.05–1.36). Concussion rates were higher in competition than practice among boys (12.04 vs. 1.46; RR = 8.27, 95% CI 6.83–10.01) and girls (9.16 vs. 1.56; RR = 5.87, 95% CI 4.69–7.34). No significant trends over time in concussion rates in boys (Fig. 2a) or girls (Fig. 2b) were observed.

### Gender comparison by injury mechanism

**Fig. 3:**
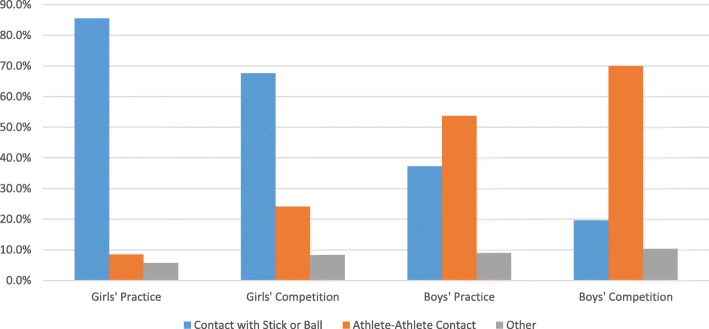
Concussion Mechanism by Gender and Type of Activity, The National High School Sports-Related Injury Surveillance Study, 2008–09 Through 2018–19. Other includes all other mechanism including contact with playing surface, contact with out of bounds object, unknown, etc

For boys’ LAX, athlete-athlete contact was the most common mechanism of concussion accounting for 66.4% of all concussions, while stick or ball contact accounted for 23.5%. In girls’ LAX, stick or ball contact was the most common mechanism of concussion accounting for 72.7% of all concussions, while athlete-athlete contact accounted for 19.8% (Table 1). The mechanism of concussion varied by gender and type of activity (Fig. 3). The rate of concussions from stick or ball contact was significantly higher in girls compared to boys overall (RR = 2.60, 95% CI 2.12–3.18), in competition (RR = 2.62, 95% CI 2.05–3.37), and in practice (RR = 2.46, 95% CI 1.75–3.50) (Table 1). Conversely, the rate of concussions from athlete-athlete contact was significantly higher among boys. This gender difference in concussion mechanisms was observed each year during the study period (Fig. 2a and b).

**Table 1:** High School Girls’ and Boys’ Lacrosse Concussion Rates per 10,000 Athletic Exposures (AEs) and Gender Comparison by Injury Mechanism. The National High School Sports-Related Injury Surveillance Study, 2008–09 Through 2018–19

	Girls’ Lacrosse			Boys’ Lacrosse			Gender Comparison		
	n (%) Rate per 10,000 AEs			n (%) Rate per 10,000 AEs			Rate Ratio (95% CI)^**a**^ Boys’ was Referent Category		
Injury Mechanism	Competition	Practice	Total	Competition	Practice	Total	Competition	Practice	Total
Contact with Stick or Ball	188 (67.6) 6.19	91 (85.8) 1.34	279 (72.7) 2.84	94 (19.6) 2.36	50 (37.3) 0.54	144 (23.5) 1.09	2.62 (2.05–3.37)	2.46 (1.75–3.50)	2.60 (2.12–3.18)
Athlete-Athlete Contact	67 (24.1) 2.21	9 (8.5) 0.13	76 (19.8) 0.77	336 (70.0) 8.43	72 (53.7) 0.78	408 (66.4) 3.09	0.26 (0.20–0.34)	0.17 (0.08–0.33)	0.25 (0.19–0.32)
Other^b^	23 (8.3) 0.76	6 (5.7) 0.09	29 (7.6) 0.29	50 (10.4) 1.25	12 (9.0) 0.13	62 (10.1) 0.47	0.60 (0.36–0.98)	0.68 (0.23–1.79)	0.63 (0.40–0.99)
Overall	278 (100.0%) 9.16	106 (100.0%) 1.56	384 (100.0) 3.91	480 (100.0%) 12.04	134 (100.0%) 1.46	614 (100.0%) 4.66	0.76 (0.66–0.88)	1.07 (0.83–1.38)	0.84 (0.74–0.95)

^a^Rate ratios compared girls’ lacrosse concussion rates to boys’ lacrosse concussion rates. 95% CI not including 1.00 indicate statistically significant differences by gender. Rate ratios greater than 1.00 indicate playing girls’ lacrosse is a risk factor while rate ratios less than 1.00 indicate playing girls’ lacrosse is a protective factor compared to playing boys’ lacrosse

^b^Other includes all other mechanism including contact with playing surface, contact with out of bounds object, unknown, etc.

Mechanisms of non-concussion head/face injuries including contusions, fractures, lacerations, avulsions, and “other” also varied by gender. For girls’ LAX, of the 44 non-concussion head/face injuries, stick or ball contact accounted for 84.1% of all injuries while athlete-athlete contact accounted for 11.4%. For boys’ LAX, of the 67 non-concussion head/face injuries, stick or ball contact accounted for 49.3% of all injuries while athlete-athlete contact accounted for 40.3%.

### Attributable risk and AR%

The attributable risk for concussion resulting from stick or ball contact associated with playing girls’ vs. boys’ LAX was 1.75 concussions per 10,000 AEs (95% CI 1.37–2.12). The AR% for concussion resulting from stick or ball contact associated with playing girls’ vs. boys’ LAX was 61.5% (95% CI 52.9–68.5). The total percent of girls’ LAX concussions that could have been prevented if girls’ LAX players wore the same helmet mandated in boys’ LAX was 44.7%. Thus, an estimated 172 of the 384 concussions reported to High School RIO in girls’ LAX during the study period could have been prevented if girls’ LAX players had been wearing the same hard shell helmet mandated in boys’ LAX.

### Comparing helmet regulation and sphere rule enforcement

The estimates presented above assume only a change in helmet regulation without any concurrent change in enforcement of the sphere rule. Theoretically, if properly and consistently enforced, the sphere rule should prevent concussions from nearly all stick strikes to the head in girls’ LAX. Thus we conducted a sub-analysis to compare the relative contribution of contact with the ball vs. contact with the stick. In girls’ LAX contact with the ball accounted for 67.9% of all concussions in practice compared to 22.3% of all concussions in competition. Contact with the stick accounted for 17.9% of all concussions in practice compared to 45.3% of all concussions in competition. The AR% for concussion resulting only from contact with the ball associated with playing girls’ vs. boys’ LAX was 68.8%. The total percent of girls’ LAX concussions that could have been prevented if girls’ LAX players wore the same helmet mandated in boys’ LAX and it only prevented concussions sustained from contact with the ball was 24.0%. Thus, even if the helmet only prevented concussions sustained from contact with the ball, an estimated 92 of the 384 concussions reported to High School RIO in girls’ LAX during the study period could have been prevented if girls’ LAX players had been wearing the same hard shell helmet mandated in boys’ LAX.

## Discussion

Both boys’ and girls’ LAX players catch a hard, rapidly moving ball using a stick with a webbed head frequently positioned near the player’s head to maintain ball possession, but substantial differences between the sports exist, with the sphere rule intended to protect against stick strikes to the head in girls’ LAX (Kelley 2018; US Lacrosse n.d.-b). Unfortunately, our observations indicate the sphere rule, as currently enforced, does not effectively prevent concussions resulting from contact with a stick in girls’ LAX. Thus, any increase in concussions sustained by stick or ball contact in girls’ LAX compared to boys’ LAX can reasonably be assumed to be related to the fact that girls are less protected from stick or ball impacts to the head because they are not allowed to wear the hard shell helmet mandated in boys’ LAX.

Based on our findings, playing girls’ LAX is a risk factor for concussion from stick or ball impacts. The rate of concussions sustained from stick or ball contact was 2.60 times higher among girls than boys. Additionally, the AR% indicates 61.5% of concussions sustained from stick or ball contact in girls’ LAX were attributed to girls not wearing the same hard shell helmet mandated in boys’ LAX. Thus, we estimate 44.7% of all concussions in girls’ LAX could have been prevented if girls were also required to wear these helmets. Even if better enforcement of the sphere rule could effectively prevent all stick strikes, when we looked only at the effectiveness of headgear in preventing concussions sustained from contact with the ball, we estimated nearly a quarter of all concussions in girls’ LAX could have been prevented if girls wore the same helmet required in boys’ LAX. Our study appears to be the first to utilize the AR%, a tool which can be used to drive targeted prevention efforts by identifying what percent of an outcome of interest can be attributed to one risk factor compared to others (Peterson et al. 2013; McKinney et al. 2017), to evaluate the effectiveness of a specific piece of protective equipment in reducing sports related injury, in this case evaluating headgear rules in high school LAX.

For nearly three decades whether the helmets required in men’s/boys’ LAX should also be required in women’s/girls’ LAX has been debated in the lay media (Pennington 2016; Miele 2017), by policy makers (Bull and Cavanaugh 2016; NFHS 2019b), and in discussion sections of peer-review publications (Lapidus et al. 1992; Harmer 1993; Diamond and Gale 2001; Otago et al. 2007; Dick et al. 2007; Lincoln et al. 2007; Xiang et al. 2014; Warner et al. 2018; Pierpoint et al. 2019a, 2019b). This ongoing debate motivated Acabchuk and Johnson to publish a manuscript which included a table titled “Relevant evidence to counter each argument against the use of helmets in women’s lacrosse” which described several arguments against helmets and, for each, provided “Evidence and/or arguments against rationale” (Acabchuk and Johnson 2017). The fact that hard shell helmets with full face masks are mandated for males demonstrates LAX sport’s US governing bodies (e.g., US Lacrosse, NCAA, NFHS) have acknowledged that male players are at risk of head/face injury, including concussion, and that they believe the hard shell LAX helmet is effective at reducing injury risk. Prior studies have demonstrated female LAX players are also at risk of head/face injury, including concussion (Marar et al. 2012; Warner et al. 2018; Barber Foss et al. 2018; Pierpoint et al. 2019a, 2019b). The main gender difference in LAX concussions is not the incidence of injury, but rather the mechanism of injury, with males most often sustaining concussions from athlete-athlete contact and females most often sustaining concussions from stick or ball contact (Lincoln et al. 2007; Xiang et al. 2014; Warner et al. 2018; Pierpoint et al. 2019a, 2019b). Although the hard shell helmets mandated in men’s/boys’ LAX have been demonstrated to be effective at reducing head injury potential from ball strikes (Rodowicz et al. 2015), these helmets are still prohibited in girls’ LAX (US Lacrosse n.d.-b). So, why aren’t females also required to wear this effective protective equipment?

“Sports culture” can be a serious impediment to athlete health and safety interventions, even when the intervention is a piece of effective and readily available protective equipment. A historical example relevant to the debate over LAX headgear is eye protection in field hockey. In 2004, the American Academy of Pediatrics and the American Academy of Ophthalmology recommended that protective eyewear be worn by all participants in sports like field hockey (AAP 2004). In the 2011–12 academic year, the NFHS mandated protective eyewear in field hockey (NFHS 2018), and research demonstrated the eyewear mandate in high school field hockey effectively reduced injury without unintended consequences (Kriz et al. 2012; Kriz et al. 2015). However other US field hockey governing bodies have resisted following the NFHS’ lead. People deeply embedded in sports culture often argue against changes in protective equipment due to fears that such measures will either change the culture of the sport, or unintentionally increase injury rates. Given the continued presence of these concerns despite a lack of supporting empirical data, we believe an evidence-based discussion surrounding the controversial issue of use of helmets in the context of girls’ LAX is warranted.

As noted above and in the introduction, epidemiologic research clearly shows girls’ LAX players sustain head/face injuries, including concussions. Further, because women’s/girls’ LAX rules mandate eyewear and mouthguard use, and as of 2017, allow the optional use of headgear meeting a new standard “developed to decrease ball-to-head and stick-to-head impact forces” (US Lacrosse n.d.-a; US Lacrosse n.d.-b), US LAX itself acknowledges that female LAX players are at risk of head/face injury and that the incidence and/or severity of such head/face injury may be reduced through use of protective equipment. It follows logically that if female LAX players are sustaining blows to the head/face that place them at risk of eye or dental injuries, these same blows to the head/face may also place them at risk of other head/face injuries, including concussion.

Our results show that allowing helmets in girls’ lacrosse would lower risk of concussion due to contact with the stick or ball. However, a real concern for public health professionals, shared by those in the sports community, is that in the process of implementing efforts to prevent one health issue, a new health issue may inadvertently be introduced or other health issues may be exacerbated. The only way to determine whether this concern is valid is to measure the prevalence of potential consequences before and after an intervention. In girls’ LAX, such research has been precluded to date because helmets are not allowed, though we can draw expectations from surrogates. For example, mandating hard shell helmet use in male LAX did not lead to an unacceptable level of unintended consequences because these helmets are still mandated, and retaining the helmet mandate if it had increased rather than decreased injury risk would have been unethical. We can reasonably conclude that introducing helmets in female LAX would likely not unacceptably increase injury risk if it did not do so in male LAX. A similar argument (that protective eyewear would actually increase injury risk) raised in opposition to the mandate of eyewear in female LAX, was proven unfounded by research showing no unintended consequences followed the eyewear mandate (Lincoln et al. 2012). Additionally, studies of the introduction of a high school field hockey eyewear mandate showed decreased injury rates without unintended consequences (Kriz et al. 2012; 2015). Finally, we can look at research on other sports such as a study of skiing and snowboarding injuries which concluded helmets clearly decreased risk of injury without any unintended consequences such as increased neck injuries (Haider et al. 2012). Although we should not expect an unacceptable increase in injuries if hard shell helmets are mandated in girls’ LAX, the only way to conclusively evaluate this concern is to introduce hard shell helmets and then monitor for unintended outcomes. If unintended consequences are observed, then hard shell helmets should be not be required.

An argument, frequently referred to as the “gladiator effect,” posits that once protective equipment is donned, athletes will feel a false sense of security and will play more aggressively and take greater risks, thus increasing injury rates. For example, the NFHS’ Soft Headgear in Non-Helmeted Sports Position Statement reads, “The use of soft headgear may produce unintended consequences, including providing a false sense of security to athletes. While a recent study shows that the use of soft headgear in soccer players did not result in an increase in other injuries, a false sense of security may result in athletes, coaches, and parents/ guardians, placing less emphasis upon other strategies that include, but are not limited to: avoidance of head impact and foul play, concussion education, and the immediate reporting of concussion symptoms.” (NFHS 2019b). There do not appear to be peer-reviewed publications reporting evidence to support this argument. Rather, a body of refuting evidence concludes there is no increase in risk taking behavior and/or injury rates associated with use of protective equipment (Lund and O’Neil 1986; Scott et al. 2007; Cusimano and Kwok 2010; Ouellet 2011; Haider et al. 2012; Ruedl et al. 2012; Brunner et al. 2015; Ruedl et al. 2019). The most pertinent examples include an RCT which found no increase in injuries, including concussion, among high school soccer players randomized to wear headgear (McGuine et al. 2019) and a study of the eyewear mandate in girls’ LAX which concluded “overall injury rates do not indicate rougher play with introduction of protective equipment” (Lincoln et al. 2012). Additionally, because hard shell helmets are still mandated in boys’ LAX, no appreciable gladiator effect resulting from their adoption must have occurred. Further, there is no evidence that females, traditionally described as playing less aggressively than males, would be expected to become more aggressive than their male counterparts should they be allowed to wear helmets. Also, if athletes truly believed protective equipment could enable them to play harder, we would expect near universal adoption of optional equipment because athletes want to play at their highest level. Yet, across sports, few athletes adopt optional protective equipment (e.g., few baseball pitchers wear head protection, few rugby players wear scrumcaps, few college field hockey players wear eyewear, and to date few female LAX players wear the newly available headgear). Finally coaches and officials have the ability to control rough/reckless play through reprimands, penalties, game ejections, and suspensions – in other words, by enforcing the rules. In short, the only way girls’ LAX players could exhibit the gladiator effect is if LAX coaches, officials, and policy makers allow them to violate the rules of play.

Finally, both sexes play with hard, fast moving lacrosse balls and sticks, yet headgear standards are vastly different. Although US LAX governing bodies have revised their rules to allow females to wear an optional piece of protective equipment (a flexible headgear), with the intention of reducing impacts due to ball and stick contact, they do not mandate their use. There is no evidence to indicate the hard shell helmet with full face mask currently mandated in boys’ LAX would not provide similar protection from stick and ball strikes in girls’ LAX, thus making irrelevant the new flexible headgear.

### Limitations

Like all studies, ours had limitations, largely associated with the data source. High School RIO captures LAX data from a convenience sample of schools with ATs and thus, our findings may not be generalizable to all US high schools with LAX teams. Concussion reporting was at the discretion of the individual AT and a uniform study definition of concussion was not provided, which may lead some to question the accuracy of the reported concussion data. However, ATs have proven to be highly reliable reporters of sports-related injuries, particularly concussions (Lombardi et al. 2016). In High School RIO, exposures are captured as number of AEs rather than hours or minutes of participation which prohibits more exact time-based injury rates. It is not reasonable to expect high school ATs, who are not present at all practices and competitions for all sports, can accurately capture minutes of practice and competition exposures for all athletes in all sports.

An additional limitation is our inability to evaluate the recent introduction of the optional flexible shelled LAX headgear on concussion rates in girls’ LAX. The headgear was first marketed in 2016, and US LAX only made it the only option for players seeking head protection in 2017 (prior to that girls’ LAX players could wear soft headgear designed for other sports). Between the product’s newness and the fact that its use is only optional, it is unclear whether or not the flexible shelled headgear is protective against concussions from stick and ball impacts. If epidemiologic studies establish that the flexible shelled headgear is effective, it should be mandatory protective equipment. To date, no evaluation of the effectiveness of this optional piece of equipment in preventing concussion has been published in the scientific literature.

Despite these limitations, this study contributes important information to the body of knowledge on the relative risk of concussion in girls’ and boys’ LAX and is novel for utilization of the AR% to evaluate concussions resulting from ball or stick impacts associated with the different gendered helmet rules.

## Conclusions

Girls’ LAX players who are allowed, but not required to wear a flexible headgear are at increased risk of concussions from stick or ball impacts compared to boys’ LAX players, who are required to wear a hard shell helmet with full face mask. Based on the AR% calculated in this study, we estimate that during the study period, 44.7% of all concussions in girls’ LAX could have been prevented if girls were also required to wear these helmets. No defendable arguments appear to exist to justify restricting female LAX players access to this effective piece of protective equipment.

## Data Availability

The dataset analyzed during the current study is available from the corresponding author on reasonable request. General lacrosse injury data can be accessed in the High School Reporting Information Online (High School RIO) Annual Summary Reports available at http://www.ucdenver.edu/academics/colleges/PublicHealth/research/ResearchProjects/piper/projects/RIO/Pages/Study-Reports.aspx (accessed on 6 Oct 2019).

## Abbreviations

LAX: Lacrosse
US: United States
AT: Athletic Trainer
AE: Athletic Exposure
RR: Rate ratio
95% CI: 95% confidence interval

## References

[CR1] AAP (American Academy of Pediatrics) Committee on Sports Medicine and Fitness (2004). Protective eyewear for young athletes. Pediatrics..

[CR2] Acabchuk RL, Johnson BT. Helmets in women’s lacrosse: what the evidence shows. Concussion. 2017;2(2):CNC39. 10.2217/cnc-2017-0005 PMC6094348 30202578

[CR3] ASTM F3137-15, Standard Specification for Headgear Used in Women’s Lacrosse (excluding Goalkeepers). 2015. https://www.astm.org/Standards/F3137.htm. Accessed 6 Dec 2019.

[CR4] Barber Foss KD, Le Cara E, McCambridge T, Hinton RY, Kushner A, Myer GD (2018). Epidemiology of injuries in women’s lacrosse: implications for sport-, level-, and sex-specific injury prevention strategies. Clin J Sport Med.

[CR5] Brunner F, Ruedl G, Kopp M, Burtscher M (2015). Factors associated with the perception of speed among recreational skiers. PLoS One.

[CR6] Bull A, Cavanaugh L (2016). Helmets: a threat to the preservation of women’s lacrosse. Strategies..

[CR7] Caswell SV, Lincoln AE, Stone H, Kelshaw P, Putukain M, Hepburn L, Higgins M, Cortes N (2017). Characterizing verified head impacts in high school girls’ lacrosse. Am J Sports Med.

[CR8] Cusimano MD, Kwok J (2010). The effectiveness of helmet wear in skiers and snowboarders: a systematic review. Br J Sports Med.

[CR9] Diamond PT, Gale SD (2001). Head injuries in men’s and women’s lacrosse: a 10 year analysis of the NEISS database. National Electronic Injury Surveillance System. Brain Inj.

[CR10] Dick R, Lincoln AE, Agel J, Carter EE, Marshall SW, Hinton RY (2007). Descriptive epidemiology of collegiate women’s lacrosse injuries: National Collegiate Athletic Association Injury Surveillance System, 1988-1989 through 2003-2004. J Athl Train.

[CR11] Haider AH, Saleem T, Billaniul JW, Barraco RD (2012). Eastern Association for the Surgery of trauma injury control violence prevention committee. An evidence-based review: efficacy of safety helmets in the reduction of head injuries in recreational skiers and snowboarders. J Trauma Acute Care Surg.

[CR12] Harmer P (1993). Protective equipment in women’s lacrosse: a conflict between the sanctity of the game and the safety of the players. Sport Health.

[CR13] High School RIO: Reporting Information Online. n.d. http://www.ucdenver.edu/academics/colleges/PublicHealth/research/ResearchProjects/piper/projects/RIO/Pages/default.aspx. Accessed 3 Dec 2019.

[CR14] Kelley C. Understanding the Women's Safety Sphere. 2018. https://www.uslacrosse.org/blog/understanding-the-women's-safety-sphere. Accessed 6 Dec 2019.

[CR15] Kerr ZY, Comstock RD, Dompier TP, Marshall SW (2018). The first decade of web-based sports injury surveillance (2004-2005 through 2013-2014): methods of the National Collegiate Athletic Association Injury Surveillance Program and High School reporting information online. J Athl Train.

[CR16] Kriz PK, Comstock RD, Zurakowski D, Almquist JL, Collins CL, d’Hemecourt PA (2012). Effectiveness of protective eyewear in reducing eye injuries among high school field hockey players. Pediatrics..

[CR17] Kriz PK, Zurakowski D, Almquist JL, Reynolds J, Ruggieri D, Collins CL, d’Hemecourt PA, Comstock RD (2015). Eye protection and risk of eye injuries in high school field hockey. Pediatrics..

[CR18] Lapidus CS, Nelson LB, Jeffers JB, Kay M, Schwarz DF (1992). Eye injuries in lacrosse: women need their vision less than men?. J Trauma.

[CR19] Lincoln AE, Caswell SV, ALmquist JL, Dunn RE, Clough MV, Dick RW, Hinton RY (2012). Effectiveness of the women’s lacrosse protective eyewear mandate in the reduction of eye injuries. Am J Sports Med.

[CR20] Lincoln AE, Hinton RY, Almquist JL, Lager SL, Dick RW (2007). Head, face, and eye injuries in scholastic and collegiate lacrosse: a 4-year prospective study. Am J Sports Med.

[CR21] Lombardi NJ, Tucker B, Freedman KB, Austin LS, Eck B, Pepe M, Tjoumakaris FP (2016). Accuracy of athletic trainer and physician diagnoses in sports medicine. Orthopedics..

[CR22] Lund AK, O’Neil B (1986). Perceived risks and driving behavior. Accid Anal Prev.

[CR23] Marar M, McIlvain NM, Fields SK, Comstock RD (2012). Epidemiology of concussions among United States high school athletes in 20 sports. Am J Sports Med.

[CR24] McGuine T, Post E, Pfaller AY, Hetzel S, Schwarz A, Brooks MA, Kliethermes SA. Does soccer headgear reduce the incidence of sport-related concussion? A cluster, randomized controlled trial of adolescent athletes. Br J Sports Med. 2019 [Epub ahead of print]. 10.1136/bjsports-2018-100238. 10.1136/bjsports-2018-100238 PMC7146941 31088784

[CR25] McKinney D, House M, Chen A, Muglia L, DeFranco E (2017). The influence of interpregnancy interval on infant mortality. Am J Obstet Gynecol.

[CR26] Miele LM. Psychology of the “Gladiator Effect” and Women’s Lacrosse. Psychology Today. 2017. https://www.psychologytoday.com/us/blog/the-whole-athlete/201708/psychology-the-gladiator-effect-and-womens-lacrosse. Accessed 1 Mar 2020.

[CR27] NFHS (National Federation of State High School Associations) High School Participation Survey Archive. 2019a. https://www.nfhs.org/sports-resource-content/high-school-participation-survey-archive/. Accessed 6 Dec 2019.

[CR28] NFHS (National Federation of State High School Associations): Soft Headgear in Non-Helmeted Sports Position Statement. 2019b. https://www.nfhs.org/media/1020195/nfhs_soft_headgear_position_statement_april_2019.pdf. Accessed 1 Mar 2020.

[CR29] NFHS (National Federation of State High School Associations) Rule Changes Affecting Risk (1982-2018). 2018. https://www.nfhs.org/media/1019586/1982-2018_nfhs_risk_minimization_rules.pdf. Accessed 6 Dec 2019.

[CR30] Otago L, Adamcewicz E, Eime R, Maher S (2007). The epidemiology of head, face and eye injuries to female lacrosse players in Australia. Int J Inj Control Saf Promot.

[CR31] Ouellet JV (2011). Helmet use and risk compensation in motorcycle accidents. Traffic Inj Prev.

[CR32] Pennington B. With headgear here, girls’ lacrosse just got safer. Or did it? The New York Times. 2016. https://www.nytimes.com/2016/10/30/sports/girls-lacrosse-headgear-concussions.html. Accessed 1 Mar 2020.

[CR33] Peterson E, Aker A, Kim J, Li Y, Brand K, Copes R (2013). Lung cancer risk from radon in Ontario, Canada: how many lung cancers can we prevent?. Cancer Causes Control.

[CR34] Pierpoint LA, Caswell SV, Walker N, Lincoln AE, Currie DW, Knowles SB, Wasserman EB, Dompier TP, Comstock RD, Marshall SW, Kerr ZY (2019). The first decade of web-based sports injury surveillance: descriptive epidemiology of injuries in US high school girls’ lacrosse (2008-2009 through 2013-2014) and National Collegiate Athletic Association women’s lacrosse (2004-2005 through 2013-2014). J Athl Train.

[CR35] Pierpoint LA, Lincoln AE, Walker N, Caswell SV, Currie DW, Knowles SB, Wasserman EB, Dompier TP, Comstock RD, Marshall SW, Kerr ZY (2019). The first decade of web-based sports injury surveillance: descriptive epidemiology of injuries in US high school boys’ lacrosse (2008-2009 through 2013-2014) and National Collegiate Athletic Association men’s lacrosse (2004-2005 through 2013-2014). J Athl Train.

[CR36] Rechel JA, Yard EE, Comstock RD (2008). An epidemiologic comparison of high school sports injuries sustained in practice and competition. J Athl Train.

[CR37] Rodowicz KA, Olberding JE, Rau AC (2015). Head injury potential and the effectiveness of headgear in women’s lacrosse. Ann Biomed Eng.

[CR38] Ruedl G, Abart M, Ledochowski L, Burtscher M, Kopp M (2012). Self reported risk taking and risk compensation in skiers and snowboarders are associated with sensation seeking. Accid Anal Prev..

[CR39] Ruedl G, Porch M, Niedermeier M, Greier K, Faulhaber M, Schranz A, Burtscher M. Are risk-taking and ski helmet use associated with an ACL injury in recreational alpine skiing? Int J Environ Res Public Health. 2019;16(17):3107. 10.3390/ijerph16173107 PMC6747234 31455037

[CR40] Scott MD, Bueller DB, Anderson PA, Walkosz BJ, Voeks JH, Dignan MB, Cutter GR (2007). Testing the risk compensation hypothesis for safety helmets in alpine skiing and snowboarding. Inj Prev.

[CR41] US Lacrosse: Women’s Lacrosse Headgear FAQs. n.d.-a. https://www.uslacrosse.org/safety/equipment/womens-lacrosse-headgear-faq. Accessed 6 Dec 2019.

[CR42] US Lacrosse: Rules. n.d.-b. https://www.uslacrosse.org/rules. Accessed 6 Dec 2019.

[CR43] Warner K, Savage J, Kuenze CM, Erkenbeck A, Comstock RD, Covassin T. A comparison of high school boys’ and girls’ lacrosse injuries: Academic years 2008–2009 through 2015–2016. J Athl Train. 2018;53(1):1049–55. 10.4085/1062-6050-312-17 PMC6333219 30451536

[CR44] Xiang J, Collins CL, Liu D, McKenzie LB, Comstock RD (2014). Lacrosse injuries among high school boys and girls in the United States: academic years 2008-2009 through 2011-12. Am J Sports Med.

